# Class 3 Obesity and Oral Health in Adults: A Scoping Review of the Challenges for Oral Healthcare Services

**DOI:** 10.3390/jcm13133856

**Published:** 2024-06-30

**Authors:** Zanab Malik, Woosung Sohn, Kathryn Williams

**Affiliations:** 1 Oral Health Services, South Western Sydney Local Health District, Campbelltown, NSW 2560, Australia; 2 School of Health Sciences (Oral Health), College of Health, Medicine and Wellbeing, The University of Newcastle, Callaghan, NSW 2308, Australia; 3 School of Dentistry, Faculty of Medicine and Health, The University of Sydney, Surry Hills, NSW 2010, Australia; 4 Nepean Blue Mountains Family Metabolic Health Service, Nepean Blue Mountains Local Health District, Kingswood, NSW 2747, Australia; 5 Charles Perkins Centre-Nepean, The University of Sydney, Surry Hills, NSW 2010, Australia

**Keywords:** Class III obesity, oral healthcare, challenges

## Abstract

**Background**: Obesity is one of the most neglected public health problems affecting both developed and developing countries. The most clinically severe obesity (Class 3 obesity) has both clinical and service delivery implications on dental services. However, associations between Class 3 obesity and oral health are minimally explored in the literature and thus poorly understood. **Aims:** This scoping review aimed to explore the existing evidence on Class 3 obesity and oral health. Methods: A literature search was performed via Medline, Scopus, Google scholar and Embase research databases. **Results**: A total of 375 papers were sourced from the database search. Twenty seven full-text papers were included in the final literature review. Results revealed findings from both quantitative and qualitative studies. Papers included results pertaining to associations with dental disease, oral health and associated behaviours, oral health-related quality of life and the barriers experienced by adults with Class 3 obesity in accessing dental services. **Conclusions**: While mixed findings were identified, this scoping review reports associations between Class 3 obesity and poor oral health across various domains including clinical parameters and oral health related quality of life. The literature has also highlighted important barriers to dental care in those with the most severe Class 3 obesity. Based upon our findings, we have summarised current oral health management implications and directions for future research.

## 1. Introduction

Overweight and obesity are defined as abnormal or excessive fat accumulation that may impair health [1]. Obesity is one of the most neglected public health problems affecting both developed and developing countries [1]. The World Obesity Atlas predicts that by 2030, 1 billion people worldwide will be living with obesity, equating to an estimated global prevalence of 17.5% [2]. In Australia, the prevalence of Class 3 obesity among adults in 2022–2023 was 4.6%, which was more than double the prevalence in 2007–2008 (2.2%), and it is increasing [3,4,5]. In the clinical setting, obesity is diagnosed when a person has a body mass index (BMI calculated as weight in kg/height in metres squared) of greater than or equal to 30 kg/m^2^, with the most clinically severe obesity (Class 3) referring to a BMI greater than or equal to 40 kg/m^2^ [6].

Obesity is a key risk factor for several chronic medical conditions, including type 2 diabetes mellitus, cardiovascular disease and hypertension [7]. It is associated with significant morbidity through poor mental health and lower quality of life, with social isolation and reduced academic achievement in childhood and employment prospects in adulthood [8,9]. As such, obesity increases the number of years lost to poor health, especially for those younger in age and in lower socioeconomic groups [9]. Given the increased risks of medical comorbidities, it is imperative that people with obesity have adequate access to, and consistently utilise, medical services [10]. Currently, the gold standard in the management of Class 3 obesity involves multidisciplinary interventions at the individual level [11]. A combination of diet, physical activity and behavioural approaches are used, in addition to pharmacotherapy and/or bariatric surgery [12].

With the rising prevalence of Class 3 obesity [4], there will be an increasing number of individuals who present with numerous complex needs relating to health, wellbeing and psychosocial impacts, all of which often complicate dental management. As such, there is a need for people with obesity to access dental services regularly; however, there is a scarcity of obesity-related dental literature available [13]. There is a complex and non-linear relationship between obesity and dental disease, and it is currently unknown if this relationship differentiates across the severity of obesity [14,15]. While the current literature base consists of cross-sectional and case–control studies, carried out with small sample sizes and thus high levels of bias, there are statistically significant small-to-moderate-magnitude correlations between obesity and chronic periodontal disease parameters [14,15]. These include disease prevalence, severity and extent, adverse outcomes to periodontal therapy, including tooth loss, and other oral conditions such as xerostomia and salivary gland hypofunction, and dental erosion [16,17]. There is also an inconclusive relationship between obesity and dental caries, with some studies reporting a significant relationship, whilst others show no association [18].

There are both clinical and dental service delivery implications of obesity [15]. Practical environmental modifications include the availability of specialised or bariatric dental chairs that can accommodate higher safe working loads of up to 500 kg, or where mobility restrictions necessitate, a wheelchair platform dental chair, bariatric bed or barouche [19]. Pre-appointment planning to accommodate physical access requirements, adjusting appointment duration and increased difficulty in carrying out dental procedures have been discussed in the existing literature [20]. Concerns for staff in the form of possible physical strain and poor posture and the risks involved in medical emergencies, delivery of dental treatment under conscious sedation and general anaesthesia have also been mentioned [15,21]. These clinical implications have mostly pertained to oral surgical procedures; however, inferences can be made for routine preventive and restorative dental management, which may be similarly challenging for the population with Class 3 obesity. 

Epidemiological studies have shown obesity is linked to greater rates of overall health service utilisation and thus increased health care expenditure, primarily due to the cost of treating associated complications and comorbidities [7,10]. Dental services have been excluded in studies examining the relationship between BMI and use of healthcare services at a population level [22]. Previous studies have confirmed poor oral health behaviours in those with obesity, including oral hygiene, dietary habits and dental service utilisation patterns [13,23]. However, the existing oral health literature minimally distinguishes the differences amongst people with Class 3 obesity, when compared to those with obesity at a lower BMI. 

This literature review aimed to explore the existing evidence on the associations between Class 3 obesity, dental disease, oral health behaviours, oral health-related quality of life and the barriers experienced by adults with Class 3 obesity in accessing dental services. The practical implications of these associations precedes a discussion of possible directions in dental care for people with Class 3 obesity and areas for further research. 

## 2. Methods

An ethics statement is not applicable because this scoping review is based exclusively on the published literature. A comprehensive literature search was performed across four relevant electronic databases: Medline, Scopus, Google scholar and Embase research databases. These databases were selected following discussion with a medical librarian for the literature relating to obesity and oral health. All published papers based on relevant topics, study participants, intervention and patient populations were included regardless of the time of publication, with the aim of answering the following research question: 

What is the existing evidence for Class 3 obesity and dental disease, oral health behaviours, and oral health-related quality of life, and what are the barriers experienced by adults with Class 3 obesity in accessing dental services? 

The grey literature was not considered in this scoping review to avoid repetition of findings from previous scoping reviews exploring adult obesity and dental care [15]. No fixed period for publication was set given the minimal available literature. Papers were excluded if they were not written in the English language, they referenced only the paediatric or adolescent population, discussed patients with obesity in general without reference to people with Class 3 obesity specifically, or they included patients with Class 3 obesity planned for, undergoing or post bariatric surgery with no baseline information for dental variables of interest. Bariatric surgery may have complicated associations and typically resulted in weight loss such that study populations would no longer be classified as having Class 3 obesity. 

The search strategy used MeSH subject headings “Class 3 obesity” or “severe obesity*” or “morbid obesity” or “clinically severe obesity” AND “oral health or disease” or “dental health or disease” or “dental caries” or “periodontal disease” or “barrier to dental or oral health” or “dental services” or “oral health care behaviours” or “oral health related quality of life.” 

**Figure 1:**
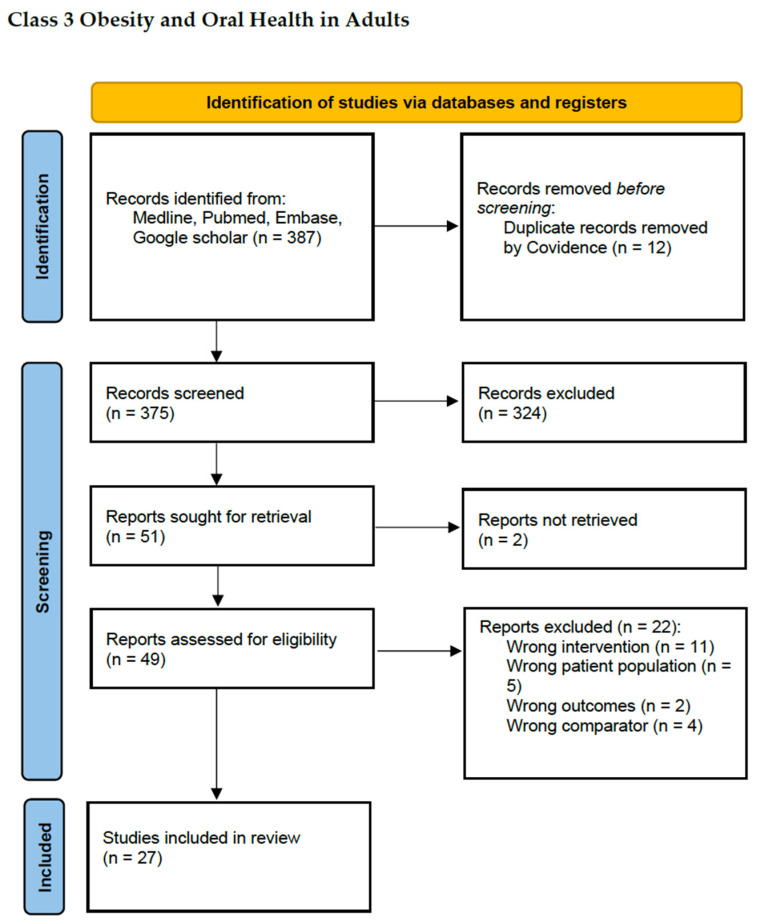
PRISMA flow diagram of the included and excluded references.

One member of the research team (ZM) performed title and abstract screening of articles. The Covidence platform was used to remove any duplicates and PRISMA (Preferred Reporting Items for Systematic Reviews and Meta-Analyses) guidelines were followed to resolve any conflicts. Full-text papers were reviewed comprehensively for inclusion/exclusion criteria and relevance to the study question (see Figure 1). The full texts were incorporated into a customised data extraction spreadsheet (see Supplement S1). A scoping review methodology was employed to explore the evidence between Class 3 obesity and oral health, given the minimal available literature, the intention to broadly collate all published research and to help establish the need for a future systematic review.

## 3. Results

A total of 375 papers were sourced from the database search. Of the 375 study abstracts screened, 324 were excluded for being irrelevant to the topic of this review, two records were not retrievable despite author request, and 22 full texts were excluded. Twenty seven full-text papers were included in the final literature review (see Figure 1). 

The included papers consisted of 19 cross sectional studies, two longitudinal studies, one case control study, one prospective study, one cohort study, one qualitative study, one review, and one case report, the majority (12/27) of which were published in Brazil [24,25,26,27,28,29,30,31,32,33,34,35], with others published in Sweden [36,37,38], Australia [39,40,41], Slovenia [42,43], France [44], Romania [45], Italy [46], Poland [47], India [48], Germany [49] and the United States of America [50]. The included papers covered both quantitative and qualitative study methods. The findings of the papers can be summarised as those relating to oral health and oral health-related quality of life and those relating to barriers and enablers to accessing dental services for people living with Class 3 obesity.

The majority of studies referenced their utilised BMI values to categorise their study populations, some of which had individuals with Class 3 obesity as the primary focus and detailed the BMI characteristics of the sample, others which also examined study groups with lower levels of obesity. Of the several studies reporting study populations with ‘morbid obesity’, individuals were variably included with BMI ≥ 35 kg/m^2^, with obesity-related comorbidities and/or BMI> or ≥40 kg/m^2^. Where the term ”morbid obesity” was used in the included studies, the authors of this review have described the study population as having Class 3 obesity to utilise a standardised term to define obesity severity and to avoid stigmatising language. Three studies included cohorts with Class 3 obesity further divided into BMI categories of increasing severity [38,39,40].

### 3.1. Oral Health and Oral Health Related Quality of Life in People with Class 3 Obesity

Oral health parameters across the various cross sectional studies assessed included dental caries, periodontal disease, tooth wear with a focus on dental erosion, number of teeth and tooth loss, variables relating to saliva, and oral health-related quality of life. 

#### 3.1.1. Dental Caries

Mixed results were found in relation to associations with dental caries in individuals with Class 3 obesity. In the Swedish study of young females aged 18–35 years by Taghat et al., 2022, the cohort of 118 participants was classified according to BMI severity (35–39.9, 40–44.9 and >45 kg/m^2^) [38]. Consistent associations between BMI and dentine caries for those with the highest BMI (≥45 kg/m^2^) were reported [38]. In contrast, the Brazilian study by Yamashita et al., 2015, reported no significant differences between individuals with Class 3 obesity and those with normal weight [35]. However, statistically significant differences were found regarding carious lesions and increasing BMI severity. They concluded that the participants with the highest BMI were most at risk of dental caries and recommended that this should be taken into consideration during planning of preventive dental visits.

#### 3.1.2. Periodontal Disease

Periodontal health was generally poor in individuals with Class 3 obesity irrespective of the severity of BMI group. Periodontal disease and associated clinical parameters assessed from the included studies in this scoping review included the assessment of periodontal probing depth, bleeding on probing, plaque index scores and clinical attachment loss [38]. Clinical examinations to assess variables were used; however, there were minor differences in the definition of periodontitis used by investigators. These were primarily assessed in groups of patients with Class 3 obesity who were undergoing bariatric surgery. 

Baseline periodontal clinical parameters revealed associations between Class 3 obesity and periodontal disease, with some studies reporting periodontitis with high severity and affecting a higher number of teeth [42], or with a high prevalence of up to 67% [43] and 70.8% [31]. A number of studies examined periodontal conditions in relation to the systemic health of patients with Class 3 obesity, including in relation to hypertension [28,42], obstructive sleep apnoea syndrome [34], incretin levels (important in glucose homeostasis) [46], metabolic-dysfunction-associated fatty liver disease and gamma-glutamyl transpeptidase levels [43]. In a study involving 111 patients with Class 3 obesity stratified into two groups: patients with (n = 54, median (1st–3rd quartile) BMI = 46.58 (42.84–50.32)) and without arterial hypertension (n = 57, median (1st–3rd quartile) BMI = 47.17 (42.81–52.53)), those with hypertension were found to have a higher prevalence of periodontitis and greater severity of periodontal disease than those without hypertension [28].

Similarly, in relation to type 2 diabetes mellitus, periodontal pocket depths and clinical attachment levels were noted to be higher amongst participants with diabetes and Class 3 obesity when compared to those without diabetes in the longitudinal study by de Moura-Grec et al., 2014 [27]. In this particular study, only 10% of 59 patients exhibited a normal periodontal condition and 45% had periodontal pockets ≥4 mm, reflecting that the majority of the study sample presented with a periodontal condition [27].

#### 3.1.3. Tooth Wear 

The included studies provided evidence for the prevalence and patterns of tooth wear described for groups of patients with Class 3 obesity. The methodology to assess tooth wear differed across studies, with different indices utilised during clinical examinations. Whilst obesity and dental erosion has been reported in the literature with a mix of cohorts of those only with Class 3 obesity and bariatric surgery cohorts, the included studies assessing tooth wear add to the evidence base on the oral health in patients with Class 3 obesity. Of the four Brazilian studies and the one German study which examined tooth wear in patients with Class 3 obesity, four of these studies focused on patients before bariatric surgery [24,25,27,35,49]. The rationale for the investigation was the potential for erosive tooth wear due to frequent episodes of gastroesophageal reflux and vomiting that can occur after bariatric surgery. However, for the purposes of this scoping review, pre-operative data were reported for prevalence and patterns of tooth wear in patients with Class 3 obesity. No studies assessed the presence of the potential associated symptoms or sequelae of tooth wear. However, this was practically explored in the single case report included in this review with clinical considerations relating to the patient’s dental erosion following bariatric surgery [50].

In the study by Alves et al., 2012 [24], the prevalence of tooth wear represented by non-carious dental lesions was reported as 35/42 (83.33%) among 42 patients with Class 3 obesity who were awaiting bariatric surgery. Prevalence was significantly higher compared with a control group of 42 individuals with normal weight (26/42, 61.90%, *p* = 0.024) who were matched on the basis of sex, smoking, educational level and ethnicity [24]. Whilst there was a significantly higher number of patients with Class 3 obesity who self-reported vomiting episodes compared with the control group (14.28% vs. 2.38%, *p* = 0.048), reflux and vomiting was not significantly associated with non-carious dental lesions in this study. Of note, the investigators reported that all patients in their study presented with some degree of tooth wear, reflective of the multifactorial aetiology of tooth wear [24]. Similarly, there was also evidence of dental erosion in a German study which included a group of 31 individuals with Class 3 obesity who had not undergone bariatric surgery [49].

Tooth wear was found to be prevalent amongst patients with Class 3 obesity (median BMI was 49.604 kg.m^2^) before undergoing bariatric surgery in the study by Aznar et al., 2019 [25]. In this study, more tooth wear was found involving anterior teeth and on the incisal and occlusal surfaces, suggesting components of attrition and/or erosive types of tooth wear [25]. This was a similar finding to that of a longitudinal study involving a group of 59 patients with Class 3 obesity with a mean (SD) BMI of 49.31 kg/m^2^ (8.75), which reported the most affected surfaces by tooth wear were the occlusal/incisal (79%), followed by buccal (8%) and lingual (13%) surfaces [27]. The study by Yamashita et al., 2015, reported increased severity of dentine wear compared with enamel wear in a group of patients with Class 3 obesity [35].

#### 3.1.4. Number of Missing Teeth, Tooth Loss and Chewing

The number of missing teeth, tooth loss, and issues related to chewing (independent of quality-of-life measures) were other variables examined across multiple studies, including in people with Class 3 obesity with mixed results. When compared with those with normal weight, one study found no significant between-group differences in relation to the number of absent teeth, bruxism and difficulty with mastication [35]. There was a similar finding in relation to tooth loss in another study [25]. In contrast, a cohort of women with Class 3 obesity noted significantly fewer teeth than those with lower levels of obesity [37]. 

A study by Godlewski et al., 2011, examined the chewing function of three groups of patients with Class 3 obesity undergoing bariatric surgery (mean BMI of sample at baseline 47.7 kg/m^2^), who presented with differences in dental status, through the assessment of chewing time, the number of chewing cycles and chewing frequency during the mastication of standardised samples of different food items [44]. Whilst changes to chewing kinematics were observed across all patients following bariatric surgery, this study did not provide any specific conclusions regarding the pre-bariatric surgery chewing function of the sample. 

#### 3.1.5. Salivary Variables 

It is possible that the risk and presentation of dental disease reported amongst those with Class 3 obesity may have been influenced by the changes in saliva, and mixed results regarding salivary variables were reported from the included studies. Among other findings, lower salivary pH [30,35], stimulated saliva flow [27,30,35] and buffering capacity [30] have been shown in studies involving participants with Class 3 obesity. In the study by de Moura Grec et al., 2014, 40.6% of 59 patients with Class 3 obesity had hyposalivation [27]. However, in another study of patients with Class 3 obesity before bariatric surgery, values of all salivary variables were within the normal range [29]. In the study by Taghat et al., 2022, involving 118 females with Class 3 obesity, no statistically significant differences were found in both stimulated and unstimulated salivary flow rates across BMI groups of increasing severity [38].

Other variables relating to saliva were reported in the literature review by Choromariska et al., 2015, which concluded from the available studies a significantly decreased salivary cortisol concentration in women with Class 3 obesity compared with those with normal weight [47,51]. Another study analysed changes in salivary proteomics, adiponectin and albumin levels in relation to weight loss and periodontitis in patients undergoing bariatric surgery; however, no conclusions were made on saliva parameters pre-bariatric surgery [32]. Hashizume et al., 2015, found elevated C.albicans levels in the saliva of patients with Class 3 obesity before they underwent bariatric surgery [29].

#### 3.1.6. Oral Health Behaviours

Several of the clinical parameters investigated across the included studies may have been influenced by oral health behaviours. However, no definitive associations relating to oral health behaviours could be established from the included studies. In the study by de Almeida Bastos et al., 2018, they investigated the prevalence of risk factors for caries, dental erosion, and periodontal disease in a group of 255 patients with Class 3 obesity referred for bariatric surgery [26]. The study sample was subdivided into two groups based on BMI severity, those with BMI ≥ 40 kg/m^2^ and those with BMI 35–40 kg/m^2^ and obesity-related comorbidities [26]. This study revealed no significant differences between the two groups with Class 3 obesity in relation to risk factors for caries, periodontal disease, and dental erosion, with similarities between the groups in oral health care behaviours, oral health characteristics, and eating habits, but not flossing frequency. However, the study reported a high prevalence of risk factors for dental erosion overall, with 78.4% of all participants reporting daily consumption of acidic foods, and 92.2% reporting daily acidic beverages. With respect to caries risk factors, 38.5% of the participants reported high sucrose intake [26].

Similarly, many participants reported dietary risk behaviours for dental caries in another study which investigated the frequency of high sugary food and drink consumption, as well as any specific dietary risk behaviours, in a cohort with Class 3 obesity [39]. This study concluded that BMI was not significantly correlated with these individual oral health behaviours (*p* > 0.05). 

Oral health habits such as toothbrushing, regular interdental cleaning, dental visiting and the reason for the last dental visit were explored in a cross sectional study of a different Swedish cohort of women with Class 3 obesity, further subdivided based on BMI severity to 35–39.9, 40–44.9 and ≥45 kg/m^2^ [38]. There were no statistically significant results in relation to these variables and BMI in this study. This was a similar finding to two recently published cross sectional studies, which also classified their cohort into BMI tertiles of increasing severity, with median (IQR) BMIs of each group of 42.3kg/m^2^ (40.3–43.2), 49.1 kg/m^2^ (47.2–51.1) and 60.6 kg/m^2^ (57.3–66.1). These studies found that BMI was not significantly correlated with oral hygiene practices, such as toothbrushing and flossing frequency [39] or dental utilisation [40]. Oral health behaviours relating to dental service utilisation included frequency of dental visits, time of last dental visit, setting of last dental visit (private/public), previous referral for dental management necessitating a bariatric dental chair, time between referral and appointment, and participant behaviour in the event of a dental emergency [40]. A key finding was the poor dental utilization by this cohort, with more than half reporting dental attendance only when needed, or for a problem, and unfavourable dental visiting patterns [40].

#### 3.1.7. Oral Health-Related Quality of Life

Mixed results from the included studies were reported in relation to oral health-related quality of life in those with Class 3 obesity; however, this was assessed using differing measures. When the Oral Impacts on Daily Performances scale was utilised among a group of Brazilian patients with Class 3 obesity to assess oral health-related quality of life, no significant differences were observed compared with subjects of normal weight [35].

In an Australian cross-sectional study involving patients with Class 3 obesity grouped into tertiles with increasing BMI severity, low oral health-related quality of life was reported by participants compared with national data [40]. However, there were no significant findings between BMI levels and the assessment of oral health-related quality of life using the Oral Health Impact Profile-14 score in this study [40].

Another Swedish paper reported differences in self-perceived oral health across various BMI groups, categorising their large cohort of men and women (total 6078 individuals) into the following groups: normal weight, underweight, Class 1, 2 and 3 obesity [36]. Oral health was assessed from a conceptual model using five specific questions to provide perspectives on oral symptoms and functional limitations including ability to chew various food types, satisfaction with the appearance of teeth, oral dryness, remaining teeth present and overall satisfaction with the mouth [36]. The group with Class 3 obesity had worse perceived chewing capacity and increased mouth dryness compared with the group with normal weight; however, no other significant differences across other variables were reported in relation to Class 3 obesity [36]. Whilst this study was limited by self-reported data of BMI and oral health care measures, it provided unique insight into the comparison of variables across differing obesity severities. 

### 3.2. Barriers and Enablers to Accessing Dental Services in People with Class 3 Obesity

A qualitative study in Australia investigated key barriers for people with Class 3 obesity (BMI range up to 84.6 kg/m^2^) in accessing dental services. The findings revealed physical environment barriers and key themes including weight-related stigma and discrimination, a lack of tailored services, unpredictability of the dental environment and a feeling of disempowerment amongst participants to act to improve their oral health [41]. Many of these findings were consistent with previously published commentary [20]. However, this was the one of three studies included in this scoping review which reflecting the unique perspectives of people living with obesity at the extreme end of the Class 3 group. Of note, enablers to dental access were also reported from this study group, including increased awareness and knowledge of obesity by providers, increased numbers of bariatric dental facilities, bulk-billed services, home visits and the use of advances in technology such as virtual care facilities [41].

## 4. Discussion

This scoping review provides an update of the dental literature reporting on the cohort with Class 3 obesity and provides a trend towards poor oral health across various domains including clinical parameters relating to dental caries, periodontal disease, tooth wear, tooth loss, salivary variables, oral health behaviours and oral health-related quality of life. Mixed findings were reported across the included studies and no clear trends were evident with increasing severity within the Class 3 obesity group. The increased literature in recent years on this topic is reflective of the growing prevalence of obesity and Class 3 obesity, which had led to greater challenges in oral healthcare and the dental environment. The collective literature in this review indicates that people with Class 3 obesity are at risk of poorer oral health and complicated dental care, although associations are not directly linked to BMI. The findings also highlight associations between oral disease and systemic markers of health within this cohort, reflecting the growing links between obesity, obesity complications/co-morbidities and oral health, with particular note made for type 2 diabetes. Dental professionals must be aware of the implications of Class 3 obesity for oral health.

The clinical implications of this review are significant and the strength of this paper is in the collation of clinically relevant findings. This review additionally provides an international snapshot of the issues relating to the oral health of patients with Class 3 obesity. The findings of several included papers clearly highlight the importance for dental professionals to emphasise preventive oral health strategies for their patients with Class 3 obesity. This includes prioritising regular dental visiting and encouraging collaboration with primary health care providers in improving the oral health and general wellbeing of people with obesity. For those at the extreme end of Class 3 obesity, additional barriers to access need to be overcome.

There were a large number of studies investigating the oral health of patients with Class 3 obesity undergoing bariatric surgery, and these patients are likely to be seen with increasing frequency in the dental setting. Dental professionals need to be familiar with the associated challenges in their dental management. Further research is needed to define the dental professional in the multidisciplinary peri- and post-operative management of the patient undergoing bariatric surgery. Whilst the focus of this scoping review was not the impact of bariatric surgery on oral health, encouragement of addressing baseline dental disease and advocating for oral health preventive measures to be implemented pre-operatively will be highly beneficial in preventing deterioration. 

Given the findings of poorer oral health and increased dental disease risk in Class 3 obesity, it is important that awareness of these associations is communicated and taught to both practicing dental professionals and students. Individuals with Class 3 obesity are more likely to have neglected oral health habits, since low self-esteem and negative self-body image both present in higher rates in this population [52], and physical access barriers are likely to impact their propensity to carry out oral health-promoting behaviours [53]. Concurrently, education in obesity management for dental professionals is reported as limited [54].

This scoping review additionally brings awareness of current access barriers for people with Class 3 obesity and enablers to accessing dental services for this group of patients. The improvement of their dental utilisation is particularly important on the background of associations with dental disease found in this review. The findings of the included papers exploring barriers to dental access suggest that patients with Class 3 obesity will require dental services that are fit for purpose. This may require referral for specialist dental management in Special Needs Dentistry departments [55]. In Australia and internationally, these departments commonly manage this cohort as they have bariatric dental chairs and are able to modify dental treatments plans when associated medical complexities are present. At a minimum, investment is required to overcome the physical environment barriers via improved access to Specialised Bariatric Dentistry Services, which may be best achieved through integration into existing medical obesity services, which has been suggested as an enabler of access for this vulnerable group [41]. This integrated multidisciplinary approach improves the emphasis of oral health behaviours as part of general health-promotion strategies for patients with Class 3 obesity, with a collective focus on obesity, oral health and nutrition [23].

The barrier of weight stigma among people with Class 3 obesity is an important finding, particularly in a group who already have barriers to accessing care, and experiences with internal and explicit stigma in other aspects of their daily lives. Weight stigma can lead to healthcare avoidance, further impairing health-related quality of life, with higher degrees of obesity associated with greater impairment [56]. Patients often report a strong sense of embarrassment following years of avoidance and feelings of self-punishment. The implications of an inadequate physical treatment space can exacerbate situations that also lead to weight stigma. 

### Study Limitations, Implications for Management and Future Research (See Table 1)

The limitation of this review was related to the majority of included papers having a cross-sectional study design, which limited the conclusions of causal associations. All included studies defined obesity based on BMI, which has known limitations [57]. Clinical parameters in the included studies were often assessed using different definitions or measures, which limited comparisons across studies. Confounding variables such as socioeconomic status, age, gender, educational level and ethnicity may have influenced comparisons between study groups and those with Class 3 obesity and were not described in detail in most cohorts.

Based upon the findings in this review, gaps in the literature include the evaluation of possible causal associations between dental disease and Class 3 obesity including factors other than BMI, the exploration of interventions to address barriers and enablers to dental access and management in this group, including physical barriers and weight stigma. 

**Table 1:** Summary of highlights for oral health management considerations and future research directions for Class 3 obesity and oral health.

Oral Health Management Implications Relating to Class 3 Obesity
Need for awareness and promotion of good dental care in those with Class 3 obesity, including through encouraging regular visits to a dental professional. Dental disease risk factors that have been identified by this review should be targeted.
Need to optimise the role of the dental professional in the management of patients with obesity, particularly Class 3 obesity. This would include integration of bariatric dentistry and obesity related education into oral health tertiary curriculum and continuing professional development courses for dental professionals.
Need to improve access to oral health care in those with Class 3 obesity, especially at the severe end, in those undergoing bariatric surgery and/or in those with medical complications such as type 2 diabetes. This could include addressing enablers to access such as increased numbers of Specialised Bariatric Dentistry Services with established referral pathways and inclusion of dental professionals in established multidisciplinary teams.
**Future Directions for Research**
1.Qualitative studies regarding: Barriers and enablers to dental access for those with Class 3 obesity from the perspective of dental professionals and health services, including physical access, medical care and weight stigma. Building capacity of dental teams to providing obesity management advice and/or defining their role as part of multidisciplinary teams managing obesity, particularly Class 3 obesity.
2.Prospective large studies examining the development and progression of dental disease, including risk factors, in cohorts with Class 3 obesity and/or those who are undergoing bariatric surgery.

## 5. Conclusions

While mixed findings were identified, this scoping review reports associations between Class 3 obesity and poor oral health across various domains, including clinical parameters and oral health-related quality of life. The literature has also highlighted important barriers to dental care in those with the most severe Class 3 obesity. Based upon the findings, we have summarised the current oral health management implications and directions for future research.

## Supplementary Materials

The following supporting information can be downloaded at: https://www.mdpi.com/article/10.3390/jcm13133856/s1, Supplement S1: Data extraction spreadsheet of included papers.
